# Enduring Effects of the COVID-19 Pandemic on the Mental Health of Physicians in Pakistan: A Mixed-Methods Study

**DOI:** 10.3390/healthcare13162009

**Published:** 2025-08-15

**Authors:** Syed Ahmed Shahzaeem Hussain, Syed Ahmed Shahzain Hussain, Muhammad Hasnain Haider, Mustafa Sohail Butt, Anas Zahid, Umair Majid

**Affiliations:** 1 Shaikh Zayed Hospital, G855+7C8 Shaikh Zayed Medical Complex، University Avenue, Block D Muslim Town, Lahore 54600, Pakistan; shahzaeemhussain@gmail.com (S.A.S.H.); shahzainhussain7@gmail.com (S.A.S.H.); 2 Liver Gut and Endoscopy Clinic, Lahore 54000, Pakistan; mhhayder168@gmail.com; 3 Hameed Latif Hospital, 14 New Lahore—Kasur Rd, Abu Bakar Block Garden Town, Lahore 54000, Pakistan; mustafasohail195@gmail.com; 4 NYC Health + Hospitals/Woodhull, Brooklyn, NY 11206, USA; anaszahidamin@gmail.com; 5 Administration, Government College University, Government College University Rd, Anarkali Bazaar, Lahore 54000, Pakistan

**Keywords:** COVID-19, SARS-CoV-2, mental health, psychological distress, mental strain, Pakistan, South Asia, mixed methods, physicians, hospital

## Abstract

**Background**: The COVID-19 pandemic caused lasting disruption to healthcare systems and the mental health of frontline workers. Though the acute crisis has passed, many healthcare workers (HCWs) continue to experience long-term psychological effects, including anxiety, grief, and burnout. This mixed-methods study investigates the enduring effects of the COVID-19 pandemic on the mental health of physicians in a low-resource country. **Methods**: Drawing on data from the ear, nose, and throat (ENT) or otolaryngology department at a tertiary care hospital in Pakistan, the study employed an explanatory mixed-methods design, combining structured surveys and semi-structured interviews. The Hospital Anxiety and Depression Scale, the Perceived Stress Scale, and the Brief COPE Inventory were administered to 42 ENT specialists, trainees, and house officers, alongside semi-structured interviews with eight ENT physicians. **Results**: Survey results revealed moderate to high levels of anxiety, depression, and stress that persisted beyond the acute crisis phase of the pandemic. Interviews provided nuanced insights into the emotional burden experienced by physicians, including persistent concerns about contagion risk, professional isolation, and increased workload. Physicians described maladaptive responses and employed active coping strategies, such as seeking peer support and utilizing adaptive problem solving. **Conclusions**: The COVID-19 pandemic has had enduring effects on the mental well-being of physicians. Targeted interventions and policy reforms that address the ongoing pressures frontline physicians face in resource-constrained environments may help mitigate these burdens, support healthcare professionals more effectively, and improve their mental health.

## 1. Introduction

The COVID-19 pandemic has precipitated profound and lasting changes in global healthcare systems and significantly impacted the professional and personal lives of healthcare workers (HCWs). While the acute crisis phase has largely abated, the residual effects of the pandemic continue to impact HCWs’ professional identities and psychosocial well-being [1,2]. The cumulative trauma associated with widespread mortality, social isolation, and economic instability has left an imprint on the healthcare workforce [3]. These enduring consequences necessitate continued scholarly inquiry, particularly as health systems seek to address the long-term ramifications on workforce sustainability, morale, and mental health.

In Pakistan, a low- to middle-income country in South Asia where this study was conducted, the number of COVID-19 cases among healthcare workers is uncertain, with no reliable published source available. Some news reports indicate that as many as 17,000 healthcare professionals were infected with COVID-19 in the first two years of the pandemic [4]. During the height of the crisis, HCWs were thrust into a context of extraordinary clinical and emotional demands [5]. They faced the pressure of managing an unwieldy patient load while grappling with a novel virus with real implications for human life [6]. Early in the pandemic, widespread bereavement emerged as a dominant theme; HCWs not only contended with the loss of patients but also with the personal grief of losing colleagues, family members, and friends [7]. The pervasive sense of loss was compounded by enforced physical distancing measures that restricted interpersonal contact and support networks. This confluence of professional and personal bereavement established a trajectory of chronic stress that, despite the periodic easing of public health threats, continues to affect HCWs on multiple levels [8,9,10]. The constant awareness of their vulnerability, coupled with the stigma associated with being perceived as potential vectors of infection, created an environment in which professional dedication was inextricably intertwined with personal risk.

The psychological scars of the pandemic are not confined to the immediate aftermath; they have evolved into a complex tapestry of risk and protective factors that continue to influence the daily lives of HCWs today, even years after the pandemic [11]. Many HCWs experienced lasting shifts in their perceptions of safety and professional fulfillment [12]. However, while research indicates that some HCWs have found resilience and adaptive coping mechanisms, others remain at risk for enduring mental health challenges, such as chronic anxiety, depression, and burnout [13]. These enduring effects highlight the importance of a nuanced understanding of the interplay between acute crisis experiences and long-term well-being. A systematic review of 42 studies conducted by researchers found that pandemics and global outbreaks in the 21st century have contributed to intense fear, anxiety, psychological distress, and depressive symptoms in individuals and communities [14].

These enduring challenges call not only for reflection but also for a targeted inquiry into the mechanisms by which pandemic-induced stressors continue to shape HCWs today, and the residual psychological, economic, and social reverberations that impact HCWs. A systematic mapping exercise of all evidence syntheses on the COVID-19 pandemic found over 202 mental health studies, of which 42 were specifically focused on the mental health of HCWs and none focused on ENT physicians [15]. ENT physicians’ daily work requires close proximity to the upper respiratory tract, where they are repeatedly exposed to potentially distressing clinical encounters and rapidly evolving procedural uncertainties as the understanding of the virus changes over time. The nature of their work, compounded by initial shortages of personal protective equipment, placed ENT physicians at the intersection of physical vulnerability and psychological strain. As such, the objective of this study is to explore these complexities and describe the protective and risk factors experienced by ENT physicians, a group of physicians who were the most directly exposed to symptomatic patients with COVID-19.

## 2. Materials and Methods

### 2.1. Approach

This study used an explanatory mixed-methods design [16,17], integrating quantitative and qualitative data collection methods to explore depression, stress, and coping among ENT physicians during the COVID-19 pandemic. The first phase (March to May 2022) involved a structured survey package consisting of three validated instruments that assessed depression, anxiety, stress, and coping strategies. Quantitative data provided an overview of prevalence within the sample. In the second phase (June to September 2022), semi-structured interviews were conducted in English with a sample of ENT physicians who participated in the survey. The goal of these interviews was to elaborate and explain quantitative results. The design was particularly important given the complexity and emotional depth of ENT physicians’ lived experiences during the COVID-19 pandemic. By using interviews to explore the “why” and “how” behind survey results, this explanatory mixed-methods approach enabled a more nuanced interpretation of the quantitative data.

### 2.2. Setting

Jinnah Hospital (JHL) is located on Usmani Rd, Quaid-i-Azam Campus, Lahore. It is located near busy neighborhoods, including Model Town, Johar Town, and Township. Jinnah Hospital is a teaching hospital with 1650 beds, associated with Allama Iqbal Medical College (AIMC) and the University of Health Sciences, Lahore. As a tertiary care hospital, it has multiple highly specialized departments (e.g., neurosurgery, pediatric surgery, and burn and reconstructive surgery) that deal with complicated cases referred from primary and secondary healthcare departments. The ENT and Head & Neck Surgery Department at the AIMC/Jinnah Hospital comprises two units with a total of 70 beds. As a large public hospital that primarily offers tertiary healthcare services free of charge, patients who visit this hospital often work blue-collar jobs and cannot afford private care. The center also provides highly specialized services, and cases from the Pakistani province of Punjab are referred to this hospital.

Given the high daily influx of patients from diverse communities, the ENT department at Jinnah Hospital serves as a crucial site for assessing both mental health risks and protective factors among ENT physicians. Renowned for its excellence in head and neck surgery, the ENT department has played a central role in managing complex ENT cases, particularly during the COVID-19 pandemic. However, the nature of these procedures placed ENT physicians at a heightened risk of viral exposure, as patients with COVID-19 often required specialized care from ENT physicians. Compounding this risk, many facilities in Lahore lack the necessary resources and equipment to perform advanced ENT interventions, resulting in the referral of more complex cases to Jinnah Hospital. This convergence of high patient volume, clinical complexity, and systemic referral patterns rendered the ENT department a critical setting for examining the intersection of occupational vulnerability and mental health. Accordingly, conducting this study at Jinnah Hospital enabled a focused exploration of how these vulnerabilities manifest in both outpatient clinics and the operating theatres/procedure rooms and impact the psychological well-being of ENT physicians.

### 2.3. Sample, Eligibility Criteria, and Recruitment

ENT physicians included ENT specialists, medical officers currently serving in the ENT department, ENT trainees, and ENT house officers (interns). ENT specialists are physicians who have completed their training and obtained one of the following ENT degrees: Fellowship in ENT (local-FCPS or foreign-FRCS, FACS), or/Master of Surgery in ENT (MS). Our sample consisted of ENT physicians with at least 1.5 months of experience in the field of ENT. This included those actively working in the ENT department at JHL at the time of the study, as well as those who had worked in the ENT department at JHL for at least 1.5 months within the previous year.

At the time of this study, there were 36 ENT physicians at the JHL: 7 ENT specialists (two professors, one associate professor, two assistant professors, and two senior registrars), 13 ENT trainees, 15 ENT house officers (interns), and 1 ENT medical officer currently working in the ENT department. Additionally, to broaden the sample size, we recruited from a sample of 12 ENT trainees and 25 house officers who had worked in the ENT department at JHL for at least 1.5 months in the year preceding this study, but were not employed by the department when this study was conducted. Therefore, the total accessible population consisted of 73 ENT physicians, trainees, and house officers. Given that the entire accessible population consisted of 73 individuals, the goal was to conduct a population-based survey rather than rely on inferential sampling that would require sample size calculations. This approach was deemed appropriate for an exploratory descriptive study aimed at understanding the breadth of mental health impacts among a well-defined and relatively small group. In such contexts, maximizing participation from the full eligible population is considered more methodologically suitable than applying traditional sample size calculations intended for generalization from a larger, undetermined population [18].

We recruited ENT specialists to complete surveys via email listserv and in-person requests, which were conducted by the research team (i.e., the first five authors), who were ENT physicians at Jinnah Hospital when the study was conducted. During the survey, participants were asked if they wanted to participate in a follow-up interview. Of the 73 ENT specialists, 42 completed the survey (57.5% participation rate).

### 2.4. Surveys

Three survey instruments were selected to inform the quantitative phase of this study:

1. The Hospital Anxiety and Depression Scale (HADS) measures anxiety and depression in a general medical population. The questionnaire comprises seven questions on anxiety and seven questions on depression [19].
2. The Perceived Stress Scale (PSS) is a 10-item instrument that measures the degree to which individuals perceive situations in their lives as stressful. It was initially developed as a 14-item instrument in 1983 by Cohen in the United States and later revised to a 10-item instrument in 1998. Initial evidence suggests that the PSS-10 may allow for meaningful comparisons across different racial, ethnic, or linguistic groups and, as such, makes it a suitable scale for use in our study, as Lahore, Pakistan, has a very diverse racial, ethnic, and linguistic composition [20].
3. The Brief COPE inventory is a 28-item self-reporting questionnaire with 14 scales, each consisting of 2 items, and can measure problem-focused, emotional, and avoidant coping styles. The COPE was structured according to Lazarus’ transactional model of stress and the behavioral self-regulation theory of Carver and Scheier [21]. Coping strategies include self-distraction, active coping, denial, substance use, emotional support, instrumental support, behavioral disengagement, venting, positive reframing, planning, humor, acceptance, religion, and self-blame [22,23].

The selection of measurement instruments and the design of interview questions in this study were guided by two models and one theory: Lazarus and Folkman’s Transactional Model of Stress and Coping [24], the Job Demands–Resources (JD-R) Model [25], and Conservation of Resources (COR) Theory [26]. The Brief COPE Inventory was used to capture coping strategies that were consistent with the Transactional Model’s emphasis on the role of cognitive appraisal and the distinction between problem-focused and emotion-focused coping. The HAD and the PSS were selected in alignment with the JD-R Model, which conceptualizes psychological strain as resulting from the interaction between occupational demands and available personal or institutional resources. COR Theory further supported the inclusion of all three instruments by highlighting the impact of actual or perceived resource loss on individual well-being, particularly in high-stress, resource-constrained environments such as the COVID-19 pandemic at a busy tertiary hospital in a low- to middle-income country. Collectively, these theories/models also informed the development of the semi-structured interview guide by shaping questions that explored physicians’ interpretations of risk, coping efforts, workplace stressors, and experiences of personal and professional loss during the pandemic.

### 2.5. Survey Analysis

Survey data for HAD and PSS were analyzed using descriptive statistics (i.e., mean) in IBM SPSS Version 30, which were compared against clinical thresholds for each survey as instructed by the authors. Survey data for the COPE Inventory were analyzed across three subscales (problem-focused, emotion-focused, and avoidant coping styles), which were then compared against clinical percentiles and normative percentiles (i.e., based on athletes) as described by the survey authors.

### 2.6. Semi-Structured Interviews

The experiences of ENT physicians during the COVID-19 pandemic cannot be fully understood through quantitative methods alone. While structured surveys offer a useful starting point for exploring mental health outcomes, they often fall short in capturing the nuanced, subjective, and context-dependent nature of experiences. In contrast, semi-structured interviews offer the flexibility for participants to articulate their thoughts, emotions, and reflections in their own words, facilitating a deeper exploration of meaning. This approach enabled the research team to gain rich insights into how ENT physicians perceived the impact of the pandemic on both their professional responsibilities and personal well-being. Through these in-depth conversations, a more comprehensive understanding emerged of the various risk and protective factors shaping their mental health throughout the crisis. The interview guide is available as an additional file.

All survey respondents were invited to participate in a follow-up interview over the phone or online. Given the sensitive and often stigmatized nature of mental health discussions among healthcare professionals in Pakistan, the aim of the interviews was not to achieve thematic saturation in the traditional qualitative sense. Instead, the priority was to include as many willing participants as possible in order to capture a wide range of experiences, perspectives, and coping mechanisms. This inclusive approach is particularly suitable for exploratory research involving small populations, where broader participation can enhance the descriptive validity of findings and provide a more complete understanding of the phenomenon under study.

Interviews were analyzed using a staged coding process informed by thematic analysis [27]. In the first stage, six researchers independently reviewed two transcripts and developed analytic memos. Then, they met to discuss their findings and collaboratively develop a preliminary coding schema that captured initial themes, subthemes, and concepts. The schema was used to create analysis templates that structured the remaining analysis. In the second stage, the remaining transcripts were independently reviewed using the analysis templates by at least two researchers each. After completing their independent coding, the pairs met to compare, discuss, and verify their interpretations. Once all transcripts had been coded, the team turned their focus to specific themes identified in the coding schema. For each theme, researchers reviewed all associated codes and developed a narrative summary. These summaries were then reviewed by all researchers. Finally, one researcher synthesized the individual summaries into a holistic representation of the findings.

### 2.7. Combining Quantitative and Qualitative Data

The inclusion of qualitative data served a vital explanatory function in conjunction with the quantitative survey findings. While the survey captured the prevalence and distribution of mental health challenges among ENT physicians, it was through the qualitative responses that deeper insights emerged into the lived realities underpinning numerical results. ENT physicians’ experiences provided context to patterns observed in the quantitative data, for example, by illuminating how factors such as professional hierarchy, personal loss, or institutional support shaped emotional responses to the pandemic. In doing so, qualitative data enriched the interpretation of quantitative data from the surveys and offered a more nuanced and humanized account of the impact of COVID-19 on the mental health and professional lives of ENT clinicians at Jinnah Hospital.

## 3. Results

### 3.1. Quantitative (Survey) Results

Of the total 73 ENT physicians and trainees at the study site, 42 responded to the survey (57.5% participation rate). Twenty (47.6%) were house officers, seventeen (40.5%) were new residents, three (7.1%) were senior residents, one (2.4%) was an Associate Professor, and one (2.4%) did not report their role. The sample included a greater population of men (n = 27, 64.3%) than women (n = 15, 35.7%). The mean age for the entire sample was 28.15 years, with a median of 27 years. All participants had an MBBS degree. In addition, five (11.9%) held an FCPS designation, two (4.8%) held an MCPS designation, two (4.8%) held a Master of Science degree, and one (2.4%) held a CMT designation. The mean number of months of medical education was 164, with a median of 96 months. The mean number of months of ENT training was 41.1, with a median of 25 months. Table 1 and Table 2 show a summary of the quantitative results, including clinical thresholds for the HAD, PSS, and Brief COPE Inventory.

### 3.2. Qualitative (Interview) Findings

Eight of the forty-two survey respondents participated in semi-structured interviews. Three of eight (37.5%) were women and five (62.5%) were men. Five (62.5%) were postgraduate residents, two (25%) were house officers, and one (12.5%) was a senior registrar/attending physician. 

During the COVID-19 pandemic, ENT physicians faced considerable stress while providing care. This study’s interview segment explored the root causes of this stress and the deeper implications behind them. Table 3 shows a summary of qualitative findings.

#### 3.2.1. The Novelty of SARS-CoV-2

The emergence of the SARS-CoV-2 virus brought uncertainty and apprehension among ENT physicians. They found themselves on the frontlines, grappling with a pathogen that was largely enigmatic at the time. The virus’s very novelty meant a dearth of comprehensive information about its origin, modes of transmission, clinical manifestations, and potential long-term effects. This knowledge gap was not merely academic; it had direct implications for clinical practice. For example, ENT physicians, often the first point of contact for patients exhibiting respiratory symptoms, acutely felt the weight of this uncertainty. Without a clear understanding of the virus’s transmissibility and manifestations, they faced challenges distinguishing COVID-19 from other respiratory ailments. This diagnostic ambiguity was further exacerbated by the lack of established treatment protocols or guidelines tailored to their specific context. In a setting where resources were already constrained, the absence of clear guidance amplified the stress and anxiety experienced by ENT physicians.

Moreover, the rapid global spread of the virus meant that information was continuously evolving. While researchers worldwide raced to understand and combat the virus, ENT physicians in this study often had to navigate this dynamic landscape with limited access to updated information and training. This intensified work-related ambiguity and placed them in a position to make critical decisions with potential life-or-death consequences, often based on limited or preliminary data.

“I couldn’t do anything because at that time, in the first wave, almost no hospital, no doctor knew how to treat the disease. And even after that, whenever I treated COVID patients on the COVID ward, in the OPDs and emergencies, I always had a kind of guilt or a kind of regret that if I knew so many things about this disease at that time, I could have handled them in a better way”.

(5095-ENT Trainee)

#### 3.2.2. Balancing Professional Exposure to SARS-CoV-2, Family Life, and Mental Health

Given the nature of their specialty, ENT physicians found themselves at a unique crossroads during the COVID-19 pandemic. The symptom profile of COVID-19 resembled that of other infectious diseases typically falling under the purview of ENT physicians. This overlap in symptoms not only introduced diagnostic challenges but also heightened the risk of exposure to the virus. As these physicians routinely performed outpatient examinations and emergency surgeries involving direct airway access, their risk of contracting the virus was significantly amplified. This direct exposure to potential sources of infection brought with it personal health risks and the looming threat of inadvertently transmitting the virus to loved ones. For example, one ENT physician asked the following:

“How will we cope with the quarantine? How will we save our kids from the infection when we come back from the COVID ward?”.

(5095-ENT Trainee)

“First is mental issues. This is your family, we have to come back. And if we get COVID, then our parents are at risk”.

(4260-ENT Trainee)

“Obviously, there is some sense of fear that my family members can get affected from me. Then…obviously you have to refrain from getting close to them”.

(2328-ENT Trainee)

As a result of heightened exposure to SARS-CoV-2, ENT physicians made the decision to distance themselves from family and friends to mitigate this risk. This self-imposed isolation, while a protective measure, had profound implications for their mental well-being. Feelings of loneliness, anxiety, and the strain of being away from their support systems exacerbated the psychological toll of their professional duties.

The emotional and psychological challenges were further compounded when their heightened risk of SARS-CoV-2 hit close to home. The fear of infecting family members was a constant source of anxiety. For some, this fear became a grim reality, leading to profound mental burnout, especially after losing a loved one. The anguish of losing a family member affected their mental health and family life and cast a shadow over their professional duties. Memories of family victims would resurface while treating patients, creating an emotional tug-of-war between personal grief and professional responsibility. Yet, the commitment to patient care remained unwavering, even amidst such personal turmoil. The ethical responsibility of treating every patient to the best of their abilities, juxtaposed with the emotional strain of personal loss, created a challenging dynamic in performing their duties.

The mandatory quarantine measures, particularly during the initial waves of the pandemic, added another layer of complexity to their challenges. After fulfilling their responsibilities in COVID-19 wards, ENT physicians were required to undergo strict quarantine, often lasting 14 days. This extended period of isolation and the inability to fulfill family responsibilities further intensified feelings of stress and helplessness. As one ENT physician shared, “In initial waves, when you have to be quarantined for 14 days after doing the COVID ward duties, how will we manage our home and kids?” (5095-ENT Trainee).

#### 3.2.3. Vaccines and Personal Protective Equipment

The onset of the COVID-19 pandemic presented numerous challenges for ENT physicians, particularly regarding vaccines and personal protective equipment (PPE). In the initial wave, the absence of a vaccine was a significant source of anxiety. The uncertainty surrounding the timeline for vaccine development and availability weighed heavily on the minds of ENT physicians:

“Initially, when there were no such hopes to have a vaccine and they were saying they will develop vaccines in 9 months or 10 months and a year or so, we were thinking will we be able to survive this long? For us, the mere diagnosis of someone having COVID felt like a death sentence”.

(7703-ENT Trainee)

When vaccines eventually became available, new concerns arose. The rapid development and deployment of the vaccine raised concerns about potential side effects. Compounding these vaccine-related anxieties was the acute shortage of essential supplies, including PPE and COVID-19 testing kits. The lack of these critical resources during the initial waves of the pandemic heightened the sense of vulnerability among physicians. Even when PPE became more accessible with time, the relief was not absolute. Physicians grappled with the lingering fear that, despite their protective gear, they might still inadvertently transmit the virus to their families and loved ones.

#### 3.2.4. Grappling with Changing Workloads to Meet Surging Demand for Healthcare

The COVID-19 pandemic led to a surge in demand for healthcare services. This, juxtaposed with a dwindling number of available ENT physicians due to infections and quarantine measures, led to a significant escalation in the workload for those still on active duty. ENT physicians found themselves managing their regular caseload and stepping in to cover the responsibilities of colleagues who were sidelined by quarantine and isolation protocols. This augmented responsibility was not limited to routine consultations. ENT physicians were often called upon to perform emergency surgeries on patients diagnosed with COVID-19. Given the inherent risks of exposure, such procedures added another dimension of stress to their already taxing roles.

The cumulative effect of these extended duties manifested in various ways. ENT physicians reported significant disruptions to their daily routines, marked by inadequate sleep, compromised dietary habits, and the constant pressure to adapt to an ever-evolving clinical landscape. The physical toll of these disruptions was palpable, but the mental and emotional strain was equally, if not more, challenging. The relentless nature of their work during this period, combined with the omnipresent threat of the virus, painted a picture of professionals striving to deliver care under exceptionally demanding circumstances.

“There are a lot of things that exaggerated our mental health issues. First, our seniors are not acknowledging our efforts while handling the COVID patients. Second, there was less human resources. Third thing, long duty hours. All these things created more mental health issues, or you can say they exaggerated our mental health issues”.

(4408-ENT House Officer)

#### 3.2.5. Navigating Medical Education and Mentorship During a Pandemic

The COVID-19 pandemic introduced unprecedented challenges to the medical training landscape, particularly for ENT trainees. A significant shift was observed in the approach to patient examinations. To minimize the risk of virus transmission, ENT trainees often abstained from conducting physical examinations, a cornerstone of their training. While this change was necessary from a public health perspective, it had unintended consequences for their educational experience. The policy of minimal clinical examinations and the closure of elective surgical operation rooms further limited hands-on learning, leading to feelings of inadequacy and incompetence among trainees. As a result of these changes, patients, accustomed to thorough evaluations, frequently voiced dissatisfaction with the omission of physical examinations. Their concerns underscored the delicate balance between ensuring safety and maintaining the quality of patient care during these trying times.

“Yes, obviously, at that time, the surgeries were stopped, the elective procedures were stopped during COVID, and our OPD was also closed for some time because of the risk factors of the pandemic, so we lost a few months [of training]”.

(8706-ENT Trainee)

Furthermore, in response to the pandemic, hospitals made significant adjustments. Elective procedures, which often serve as valuable learning experiences for trainees, were largely deferred. Even emergency procedures, typically prioritized, were conducted at a reduced capacity due to logistical challenges and the overarching need to minimize potential virus exposure. Furthermore, duty hours were curtailed to mitigate the risk of cross-infection, and the number of on-duty doctors was reduced.

A notable ripple effect of the pandemic was observed in the behavior of senior ENT physicians. Driven by the reduced procedural capacity in hospitals and genuine apprehension about the risk of infection, many seasoned ENT professionals opted to limit their hospital visits. Their reduced presence meant that junior physicians and trainees missed out on the invaluable mentorship and hands-on learning opportunities typically provided by their more experienced counterparts.

#### 3.2.6. Stigma, Social Support, and Societal Pressures as an ENT Physician During a Pandemic

The COVID-19 pandemic posed direct health threats and unveiled many psychosocial challenges for ENT physicians, particularly in social support and stigma. A glaring concern expressed by ENT physicians was the conspicuous absence of structured mental health programs or support mechanisms from governmental bodies or their hospital administration. In this void, many physicians found solace in their senior colleagues, turning to them as a source of guidance, understanding, and shared experience. One poignant account from an interviewee, the first in his department to be tasked with collecting COVID-19 samples during the initial wave, captures the depth of this emotional turmoil:

“The day I got the news, I was so depressed…I called my head of department…But he said…we cannot retreat”.

(7703-ENT Trainee)

However, this reliance on senior physicians was not universally beneficial. Some junior ENT physicians felt that their seniors were dismissive of their mental health concerns, perceiving them as mere excuses to avoid responsibilities. This lack of acknowledgment and validation of the juniors’ efforts and struggles exacerbated their stress.

The initial wave of the pandemic saw the implementation of stringent Standard Operating Procedures (SOPs) to curb the spread of the virus. Yet, these SOPs, particularly those involving the use of PPE, posed significant challenges for physicians. The discomfort of wearing PPE in hot weather conditions was palpable, as one physician recounted:

“During initial waves we used to wear so much of the protective equipment… so you can like have dehydration or sweating problem… but we have worked in very much humid and suffocating type of wards…”.

(5095-ENT House officer)

Compounding these challenges were patients’ observed non-compliance with SOPs and a perceived lack of support from hospital administrators. Patients often turned to social media for information about the pandemic, placing trust in videos they encountered. This reliance on social media and a general lack of understanding about the pandemic made many patients resistant to physician advice, leading to heightened anxiety among ENT physicians:

“Despite hospital efforts to prevent overcrowding, there was a significant communication gap between the administration and the patients. There was a noticeable lack of patient education, and many disregarded the importance of wearing masks. They would frequently visit the hospital, often accompanied by multiple family members, including children. This behaviour contributed to the spread of infection among many doctors working there.”

(8706-ENT House officer)

Beyond the immediate clinical setting, ENT physicians grappled with societal challenges. A pronounced stigma was associated with testing positive for COVID-19. This stigma extended beyond the individual to their families, making it challenging to communicate and counsel even close relatives about the nature of the disease:

“I was not able to counsel my families that this is not a stigma, this is not a taboo, we have to deal with it, but they were of the opinion, we cannot share it”.

(7703-ENT Trainee)

This societal stigma, combined with the challenges faced in the clinical setting, created a milieu where ENT physicians felt increasingly isolated. The weight of this stigma deterred many from seeking the very social support that could have been instrumental in alleviating their distress.

## 4. Discussion

### 4.1. Summary of Study Findings

Our study explored the mental health of ENT physicians who served during the COVID-19 pandemic at a tertiary, public hospital in Pakistan, utilizing both survey instruments and semi-structured interviews. The survey results revealed that over three-quarters of participants (78.6%) met the clinical threshold for abnormal levels of anxiety and depression, while an additional 14.3% fell within the borderline abnormal range. Stress levels were similarly concerning, with 81% of respondents reporting moderate stress and 11.9% reporting high stress, according to the PSS. These findings suggest a significant mental health burden among ENT physicians even after the acute phase of the pandemic was over.

Coping assessments based on the COPE Inventory indicated a moderate reliance on problem-focused and emotional coping strategies, with scores closely aligning with normative percentiles. Notably, avoidant coping remained below the midpoint, suggesting that while psychological distress was high, most physicians did not rely primarily on maladaptive strategies. Qualitative interviews added further depth to these findings, revealing specific stressors unique to ENT practice during the pandemic: heightened exposure risk from aerosol-generating procedures, isolation from family, rapidly changing clinical protocols, and inadequate access to PPE. Additional burdens included disrupted medical training, increased workloads due to staff shortages, and limited availability of mental health services. At the same time, physicians reported disruptions to medical training, extended workloads due to colleagues’ quarantines, and difficulties in accessing mental health support. These combined findings underscore the persistent burden on ENT physicians and raise broader questions about how global health systems can better support frontline providers in the long term, even after a crisis subsides.

### 4.2. Comparing Survey and Interview Findings

Survey findings revealed that ENT physicians experienced substantial emotional distress during the COVID-19 pandemic. The mean scores for anxiety and depression fell well within the clinically abnormal range, while perceived stress levels reflected a sustained sense of psychological strain. Although these standardized measures offered important numerical insights into the prevalence of mental health challenges, they could not, on their own, convey the full depth and complexity of physicians’ lived experiences. The qualitative interviews helped illuminate the emotional narratives behind the data, offering critical context for understanding what the numbers signified in real-world terms. For instance, while many participants reported moderate to severe anxiety on the surveys, their interview accounts revealed specific emotional drivers: a pervasive fear of inadvertently transmitting SARS-CoV-2 to family members, distress over witnessing colleagues fall ill, and guilt stemming from feelings of clinical inadequacy in the face of an unpredictable and evolving disease. These emotions were compounded by material stressors, such as limited access to personal protective equipment and the continuous adaptation to shifting treatment protocols. Together, these insights reveal how the psychological burden reported in surveys was deeply intertwined with the practical and ethical challenges physicians navigated daily. These findings echo patterns observed in both qualitative and quantitative research on healthcare providers in diverse global settings [31,32,33,34,35,36].

Moreover, survey findings suggested that ENT physicians predominantly relied on active coping strategies, with scores in the problem-focused and emotional coping domains falling within normative clinical percentiles. These approaches—such as adaptive problem-solving, seeking emotional support, and maintaining professional and social networks—appeared to serve as protective factors that buffered against more severe psychological deterioration. However, interview data offered an important context by revealing the limitations of these strategies. Physicians frequently described depending on informal support systems (i.e., colleagues, friends, and family) rather than on formal or institutional mental health resources. While these interpersonal connections were vital in the moment, their informal nature also highlighted a critical gap in the structured, accessible psychological support available within the healthcare system.

Importantly, both survey and interview findings highlight ongoing challenges that extend beyond the initial COVID-19 waves. Physicians spoke of lingering anxiety around residual cases, pandemic-related financial strains on healthcare systems, and the long shadow cast by collective trauma. This combination of numerical breadth and narrative depth illuminates the multifaceted nature of the mental health outcomes of ENT physicians.

### 4.3. Reflections on Provider Mental Health in the Post-Pandemic Era

As global research attention has shifted away from the COVID-19 pandemic, the psychological ramifications for healthcare providers remain undeniably salient. Although the acuity of the crisis has abated, our findings suggest that frontline clinicians carry lasting emotional burdens shaped by prolonged exposure to traumatic events and moral dilemmas. The fear of transmitting the virus to loved ones, which drove many to self-isolate or reduce contact with family, has given way to an enduring sense of vigilance and, in some cases, guilt. Providers who lost close relatives or colleagues to COVID-19 exhibit signs of moral injury, wrestling with questions of professional responsibility and personal grief [33,37,38,39].

These outcomes parallel the broader global phenomenon of “post-pandemic burnout” [32,40,41], wherein healthcare systems strive to return to normalcy even as their workforces grapple with lingering stress and self-doubt. This burnout is not solely a byproduct of overworking during the pandemic; it is compounded by the recognition that supportive structures (e.g., mental health services, peer counseling, and accessible debriefing sessions) continue to be insufficient. Even institutions that offered temporary relief measures during the COVID-19 pandemic have not always transitioned these into permanent resources, given that health systems are facing extraordinary fiscal austerity around the world. Majid et al. conducted an umbrella review of studies on the mental health of care providers during the pandemic and found insufficient organizational support to be among the most salient factors adversely affecting mental health during the pandemic [14]. This included weak control over working conditions, loose organizational structures, lack of communication and psychological support, unclear job instructions, and a sense of being blamed by management [14].

Moreover, the cyclical nature of outbreaks—exemplified by seasonal waves and new variants—has perpetuated a background level of anxiety. ENT physicians in the present study, whose roles involve high-risk airway procedures, continue to confront concerns regarding infection control and clinical safety protocols. Their sense of readiness for future pandemics can coexist with skepticism about institutional preparedness, creating ambivalence toward the adequacy of lessons learned.

This collective experience calls for a reconceptualization of provider mental health as a long-term strategic investment rather than a transient crisis intervention. For this to happen, psychosocial support services must recognize the cumulative toll of repeated exposures to adversity while also engaging healthcare workers in restoring trust in public health structures. A sustained global focus on mental health, bolstered by well-funded research, pragmatic policies, and inclusive interventions, will be essential to safeguard the resilience of providers.

### 4.4. Implications for Medical Training and Education

Our study highlights that the COVID-19 pandemic disrupted the continuum of medical education for ENT trainees in significant ways, with potential ripple effects on healthcare capacity in the years to come. Hospitals inadvertently compromised clinical exposure by limiting elective procedures and direct patient examinations, which are core pillars of medical learning. This shift, while necessary to curtail virus transmission, left some trainees feeling ill-prepared for the procedural and diagnostic demands of specialty practice. Moreover, physicians, aiming to reduce their own exposure risk, reduced their in-hospital presence, thereby reducing mentorship opportunities available to trainees. Although digital solutions such as tele-mentorship, webinars, and virtual case discussions provided interim alternatives, many trainees in this study, as well as in other studies, reported struggling to acquire hands-on skills essential for surgical practice [42,43,44,45].

These findings suggest a pressing need for robust educational frameworks that withstand crises. First, institutions must develop structured “crisis curricula” that adapt core competencies for remote and distanced learning environments through simulated training modules, augmented reality procedures, or supervised virtual consultations. Second, mentorship strategies should incorporate blended learning models, ensuring that trainees maintain access to senior physicians through virtual platforms, even if in-person contact is limited.

Additionally, the psychological well-being of trainees warrants specialized attention. Residual stress, combined with perceived academic setbacks, can fuel burnout early in a career, diminishing long-term clinical acumen and job satisfaction [46]. Academic institutions and teaching hospitals should incorporate regular mental health check-ins, confidential counseling services, and peer support networks into their curricula. Implementing these measures would not only address trainees’ immediate educational gaps but also model a more holistic and resilient approach to medical training that can endure public health emergencies.

### 4.5. Implications for Institutional Policy and Public Health Practice

Institutional policy must adapt to the lessons learned from this pandemic, prioritizing resource allocation, workload management, and cohesive support structures for healthcare providers. Our findings underline the potentially destructive impact of resource scarcity on physicians’ mental health, particularly among specialists whose scope of practice places them at an elevated clinical risk. In the earliest waves of COVID-19, ENT physicians were disproportionately affected. The nature of their work—performing aerosol-generating procedures such as nasopharyngoscopy, tracheostomies, and upper airway suctioning—placed them at the frontline of exposure. Compounded by limited supplies of PPE and inadequate testing capacity, these conditions produced an acute sense of vulnerability and moral distress among ENT physicians.

A key policy takeaway is the imperative of stockpiling, or at least streamlining access to, critical supplies so that healthcare systems can swiftly mobilize resources during crises. ENT physicians’ experiences during COVID-19 vividly illustrate how lapses in logistical preparedness can translate into real psychological harm and impaired service delivery. However, beyond material resources, equitable distribution of workload is essential to sustain specialist services during periods of sustained system stress. As several ENT physicians in this study described, surging patient volumes and repeated staff quarantines created scenarios where a small pool of clinicians was repeatedly overburdened. These extended duty hours, coupled with constant exposure risk, intensified psychological strain and threatened continuity of care.

Formal mechanisms, such as rotating on-call systems and integration of multidisciplinary support teams, can help mitigate burnout by ensuring healthcare workers—particularly those in high-risk specialties—are not continuously overextended. Additionally, institutions must prioritize transparent and timely communication during health emergencies. Several ENT physicians noted inconsistent messaging or a lack of input into operational decisions eroded trust and amplified feelings of professional isolation. Clear communication pathways and inclusive decision-making processes help anchor staff morale and cohesion, especially in times when fear, uncertainty, and rapid policy shifts are the norm.

Policy efforts must also emphasize mental health infrastructure for providers. Current mental health interventions are too often ad hoc, leaving many reluctant to seek help due to stigma or scheduling constraints. Integrating routine psychological support (e.g., such as check-ins, peer support groups, and confidential counseling) into the organizational culture can normalize conversations about mental well-being and facilitate early interventions [14,47]. Furthermore, public health agencies can develop centralized reporting mechanisms to identify emerging trends in provider stress levels, ensuring that institutional responses are timely and adequately funded. Governments and professional bodies can streamline guidelines at the macro level, reducing confusion for specialties like ENT that grapple with rapid changes in recommended protocols. By consolidating, updating, and disseminating best practices in a timely manner, policymakers can equip clinicians with consistent and accurate information.

### 4.6. Limitations and Strengths of This Study

First, our sample consisted predominantly of junior physicians, which potentially limits the generalizability of the results to more senior or experienced physicians. Second, the study relied on self-report data, which can introduce response bias due to perceived social desirability or recall inaccuracies. Finally, the sample was drawn from a single institutional context, which may raise questions about its transferability to other regions or specialties.

Despite these caveats, our mixed-methods study quantified the extent of stress, anxiety, and depression and delved into contextual nuances. By focusing on an ENT department—a specialty with inherently higher exposure risks during respiratory pandemics—our findings highlight the specific vulnerabilities and coping strategies of healthcare providers on the front lines. Ultimately, our research contributes to a growing body of global literature examining the long-term and enduring mental health implications of pandemics for healthcare providers, offering insights applicable to public health emergencies across diverse healthcare systems.

## 5. Conclusions

The findings of this mixed-methods study indicate that the mental health impact of the COVID-19 pandemic on ENT physicians—including anxiety, depression, stress, and coping styles — extends well beyond the acute crisis, since this study was conducted right after the last crisis phase ended in Pakistan, underscoring enduring threats to emotional well-being, career development, and overall resilience. As global discourse shifts away from the pandemic, healthcare institutions, policymakers, and medical educators must draw on these lessons to implement evidence-based strategies that address ongoing health challenges. Future research should continue to explore the enduring impacts of different types of healthcare providers, including how their roles, social locations, and hospital environments influence mental health outcomes. Notwithstanding, recognizing that the distress of frontline providers persists in the post-pandemic era can provide knowledge and tools to strengthen global health systems and better safeguard the professionals so vital to public health for emergencies yet to come.

## Figures and Tables

**Table 1:** Summary of quantitative results.

Survey	Results	
Anxiety and Depression: The Hospital Anxiety and Depression Scale (HAD)	Mean (SD)	1.30 (0.54)
	95% CI	(1.13, 1.47)
	Range	0.29–2.57
	Median	1.39
Stress: Perceived Stress Scale (PSS)	Mean (SD)	2.18 (0.61)
	95% CI	(1.99, 2.37)
	Range	0.60–3.60
	Median	2.15
Coping: COPE Inventory	Mean (SD)	2.14 (0.40)
	95% CI	(2.02, 2.26)
	Range	1.5–2.93
	Median	2.09
Coping: COPE Inventory (Problem-Focused Coping)	Mean (SD)	2.35 (0.63)
	95% CI	(2.17, 2.56)
	Range	1.25–3.88
	Median	2.38
Coping: COPE Inventory (Emotion-Focused Coping	Mean (SD)	2.40 (0.59)
	95% CI	(2.21, 2.58)
	Range	1.50–3.67
	Median	2.36
Coping: COPE Inventory (Avoidant Coping)	Mean (SD)	1.85 (0.45)
	95% CI	(1.72, 1.99)
	Range	1.25–3.00
	Median	1.73

**Table 2:** Comparing survey results to clinical thresholds.

Survey	Clinical Thresholds	Survey Results
Anxiety and Depression: The Hospital Anxiety and Depression Scale (HAD) [28] Mean: 18.10	0–7: Normal	n = 3 (7.14%)
	8–10: Borderline abnormal	n = 6 (14.29%)
	11–21: Abnormal	n = 33 (78.57%)
Stress: Perceived Stress Scale (PSS) [29] Mean: 21.43	0–13: Low stress	n = 3 (7.14%)
	14–26: Moderate stress	n = 34 (80.95%)
	27–40: High stress	n = 5 (11.9%)
Coping: COPE Inventory * [30]	Problem Focused Coping	Normative Percentile: 59.2 Clinical Percentile: 52.7
	Emotional Coping	Normative Percentile: 62.4 Clinical Percentile: 59.4
	Avoidant Coping	Normative Percentile: 35.9 Clinical Percentile: 39.6

* COPE Inventory analysis is based on clinical percentiles and normative percentiles based on athletes as reported by the inventory authors [29].

**Table 3:** Summary of qualitative findings.

Theme	Description	Illustrative Quote
The novelty of SARS-CoV-2	ENT physicians faced immense uncertainty due to the new and poorly understood nature of the virus. They lacked access to up-to-date information, clear treatment protocols, and training, all of which amplified clinical and emotional stress.	“I couldn’t do anything because at that time, in the first wave, almost in no hospital, no doctor knew anything, how to treat the disease. And even after that, whenever I treated COVID patients on the COVID ward, in the opds and emergencies, I always had a kind of a guilt or a kind of regret that if I knew so much things about this disease at that time, I could have handled them in a better way” (5095-ENT Trainee).
Balancing Professional Exposure to SARS-CoV-2, Family Life, and Mental Health	ENT physicians experienced the emotional toll of being high-risk frontline workers, fearing infecting loved ones, and undergoing repeated quarantine. Isolation and loss of family members contributed to heightened stress and emotional exhaustion.	“how will we cope with the quarantine, how we’ll save our kids from the infection when we come back from the COVID ward?” (5095-ENT Consultant).
Vaccines and Personal Protective Equipment	Anxiety surged due to the initial lack of vaccines and personal protective equipment (PPE). Later, fears about vaccine side effects and inadequate protection despite PPE use persisted, sustaining stress levels among ENT physicians.	“Initially, when there were no such hopes to have a vaccine and they were saying they will develop vaccines in 9 months or 10 months and a year or so, we were thinking will we be able to survive this long? For us, the mere diagnosis of someone having COVID felt like a death sentence” (7703-ENT Trainee).
Grappling with Changing Workloads to Meet Surging Demand for Healthcare	Increased workload due to infected or quarantined colleagues led to mental and physical exhaustion. Lack of recognition from senior staff and long duty hours compounded burnout and mental distress.	“There are a lot of things that exaggerated our mental health issues. First, our seniors not acknowledging our efforts while handling the COVID patients. Second, there was the…less human resources. Third thing, long duty hours. All these things created more mental health issues, or you can say they exaggerated our mental health issues” (4408-ENT House Officer).
Navigating Medical Education and Mentorship During a Pandemic	Training disruptions due to closed clinics, reduced surgeries, and absentee senior mentors left ENT trainees feeling incompetent and unsupported. Reduced learning opportunities hindered skill development and contributed to stress.	“Yes, obviously, the, at that time, the surgeries were stopped, the elective procedures were stopped during COVID, and our OPD was also closed for some time because of the risk factors of the pandemic so we lost a few months” (8706-ENT Trainee).
Stigma, Social Support, and Societal Pressures as an ENT Physician During a Pandemic	Lack of institutional mental health support, stigma around COVID-19, and lack of compliance with standard operating procedures (SOPs) contributed to emotional isolation. Conflicts with hospital administration and public misinformation further strained ENT physicians.	“The day I got the news, I was so depressed…I called my head of department…But he said…we cannot retreat” (7703-ENT Trainee).

## Data Availability

The original contributions presented in this study are included in the article. Further inquiries can be directed to the corresponding author.
